# Early Detection and Intervention: A Framework for Preventing Academic Failure in Medical Students

**DOI:** 10.12669/pjms.41.3.10883

**Published:** 2025-03

**Authors:** Azhar Rashid, Rahila Yasmeen, Rehan Ahmed Khan

**Affiliations:** 1 Azhar Rashid, Dean Faculty of Health and Medical Sciences, Principal, Islamic International Medical Collage, Al-Mizan Campus, Riphah International University, Rawalpindi, Pakistan; 2 Rahila Yasmeen, Director Offices of Research, Innovation and Commercialization, Dean The Riphah Academy of Research and Education, Islamic International Medical College, Riphah International University, Rawalpindi, Pakistan; 3 Rehan Ahmed Khan, Dean Riphah Institute of Assessment, Professor of Surgery, Head of Department, Islamic International Medical Collage-Trust, Riphah International University, Rawalpindi, Pakistan

**Keywords:** Academic Failure, Framework, Predict, Pakistan, Undergraduate medical student

## Abstract

Academic failure is multifactorial with personal, institutional, and societal factors. Identification after high stake assessments comes too late for meaningful interventions. There is limited data to predict academic failure at an early stage. This qualitative exploratory study aimed to identify such students using a predictive framework. Using purposive sampling, twenty-seven participants (8 academic failures, 8 high achievers, and 7 medical teachers) were enrolled after informed consent and ethical approval. One-to-one interviews with eight academic failures and two focused group discussions (FGDs), one with the high achievers and the teachers were conducted online using a validated questionnaire. Thematic analysis and blended coding was done manually with member checking and triangulation. Key predictors included poor self-regulation, procrastination, emotional imbalance, low self-efficacy, cognitive overload, and non-reflective practices. Key components of the framework suggested are documentation of students’ prior academic results and professional choice, at the time of admission as well as behavioral traits and performance in formative assessments. Close observation of procrastination, emotional state, and self-efficacy during small group discussions by teachers followed by feedback will identify students at risk. Reflective practices by both students and teachers during feedback sessions will uncover hidden learning gaps. In conclusion, “potential academic failures” can be identified and supported by observation, documentation, and reflective practices by teachers and students using proposed framework.

The undergraduate students in Pakistan face a tough competition to qualify for the entry test requirements in the medical colleges. In 2023, more than 180,000 students took the Medical and Dental Colleges Admission Test (MDCAT) in Pakistan.^1^ Upon qualifying to enter a medical school, marks beginning of a new journey. The undergraduate medical education is challenging. The early prediction of a potential academic failure is crucial. After the failure in the first major assessment, the retrieval of the student becomes very difficult.^2^ Pinyopornpanish et al have classified the factors leading to academic failure in preclinical years as personal, institutional, and social. Before admission these include poor academic scores, inadequate planning and regulation, low motivation and self-efficacy, and poorly designed curricula.^3^,^4^ Yates has created a toolkit to identify preclinical students who may not succeed based on failing three or more exams annually, poor health or social challenges, delayed Hepatitis-B vaccination and negative attitude comments by faculty.^5^ The study was conducted at Islamic International Medical College from March 2021 to August 2021 where an integrated, modular and student-centered curriculum is in practice.

There is a gap in the literature regarding a framework in undergraduate medical students to predict academic failure before the first high stakes assessment. We report a framework for early detection of academic failure, developed from an exploratory qualitative study.

A purposive and maximal variation sampling approach was employed to select twenty-three participants, including eight academically struggling students, eight high achievers, and seven experienced teachers. Students failing maximum examinations, four each from fourth and final year who consented for the study were included in the academic failure group while high achievers in the study included top four position holders, four each from fourth and final year. Medical teachers of IIMC with minimum five years teaching experience and holding diploma or masters in health professions education were included in the teachers’ group. One-to-one interviews with eight academic failures (45 minutes), two focused group discussions (FGDs) one each with the high achievers and the teachers of 90 minutes duration were conducted on MS teams. A total of 16 articles were selected after screening 70 records using PRISMA 2009 guidelines, with quality scores ranging from 25% to 91% (average 66%). Themes were developed after piloting and final interview and FGD questions were prepared by combining findings from the literature review and pilot study. Data was collected using semi-structured, open-ended questionnaires. Thematic analysis was performed with manual transcription and coding. Member checking and triangulation was done to ensure validity and reliability of data.

## Ethical Approval:

The study was conducted after approval by the Riphah International University Ethical Review Board (Ref# Riphah/IRC/21/10, Date: February 16, 2021)

The first objective of this study was to identify the factors which could predict academic failure before the first assessment, combined block assessment (CBA) in our setup. The first CBA is four months after the admission and before that there are 11 low-stake/formative assessments. The factors identified were poor past academic record, lack of self-regulation, procrastination, hesitant help-seeking, and cognitive overload. Personality and psychological makeup included lack of motivation, introversion, and poor emotional health. Additionally, the assessment of social context was conducted but proved inconclusive in our study published earlier.^6^ We report here the second objective of this study that was to develop a framework for the early detection of academic failure that was observable, measurable and could be documented based on the factors identified. A framework is a structured guide, for achieving the objective.

In medical education, Miller’s Pyramid, and Bloom’s Taxonomy are examples of frameworks for preparing a curriculum while the research framework is made from the theories to provide a frame for the research, called theoretical framework.^7^ The advantage of a framework is that it provides a focused and directed path to reach the objective from the ideas and categories evolved from a study. The cue for preparing the framework has been taken from the article by McMeekin et al on developing methodological framework.^8^ The framework is shared in Table-I, and has four main components, derived from the themes except interactive reflective feedback which was a subtheme.

**Table-I:** Framework for early detection of Academic Failure.

Sr. No.	Main components of framework	Factors for observation measuring and documentation	Process to complete the task
1	Educational Journey	Previous Results of SSC / ‘O’ levels, HSSC / ‘A’ levels, MDCAT or equivalent defined by the regulatory body of the country Coaching for studies Choice of profession – own will or not	To be documented on admission in the medical college and kept in the student’s branch.
2	Behavior	Procrastination, emotional state, Introvert, low self-efficacy	To be observed and documented by the teacher in the SGDs, PBLs and mentoring group sessions
3	Task Performance	Performance in the low stake / formative assessment	Record of the marks will be kept in the student’s branch Comments on the performance will be written and documented by the examiner and identify/label potential academic failure.
4	Interactive reflective practice	Reflection on the performance in the low stakes / formative assessment by the student and the teacher in feedback session	Reflection by the student and the teacher in the low stakes will surface the hidden or unknown gaps in learning and will facilitate in the early detection of the academic failures. Summary of reflection by the student and the examiner will be documented by the examiner and label a “potential academic failure/students requiring additional support”

**SSS:** Secondary School Certificate, **HSSC:** Higher Secondary School Certificate, **MDCAT:** Medical and Dental College Admission Test, **SGD:** Small Group Discussion, **PBL:** Problem based Learning, (The class teacher will prepare a list of the “potential academic failure/students requiring additional support” to be reviewed by a committee under the chairmanship of the principal to include Director Academics and relevant head of departments).

This proposed framework can be used for both initial and continuous monitoring of a student’s overall performance in a medical school. In our set up CBA is considered as a major assessment however it can be used for any first major assessment. A student enters the medical school with a past academic record and a learners skillset acquired over past educational years which can be documented. Multiple small group discussions and low stake/formative assessment are held before the first CBA. It provides ample opportunity for the teacher to observe, measure and document students’ ability and behavior for filtering a potential academic failure.

Interactive reflective feedback in the formative assessments can further help in reaching the objective and can improve learning. It was starkly deficient in our study. It has been included as a separate domain in the framework. It is a metacognitive activity. According to Hayat AA et al., students’ learning-related emotions and metacognitive methods are influenced by their sense of self-efficacy, and this in turn affects their academic achievement.^9^ The facilitator can assess cognitive ability, behavior and psychological make-up of the student, that can further enhance self-efficacy and learning. Our findings serve as a basis for unifying various existing concepts into a framework and help address them cohesively. A recent study by Morosanova et al explored the impact of key non-cognitive factors on academic success. The findings showed that intentional self-regulation is essential for raising students’ motivation, engagement, and favorable attitudes about learning in addition to their academic achievement suggesting an approach, integrating various factors.^10^

Students can be counselled on areas identified by this evidence-based framework early on so that their challenges are addressed. The objective assessment will help the struggling students to identify their weak areas which need improvement as well as a holistic model to raise lifelong learners.

## Limitations of this study:

The findings may be specific to a single institution with a modular curriculum. However, the framework is designed to be adaptable, enabling its application across various curricular settings. Challenges like low task performance, procrastination, and lack of reflective practices are widespread in medical colleges throughout Pakistan, not limited to modular curricula. The framework aligns with the objectives of regulatory bodies such as the Pakistan Medical and Dental Council (PMDC), focusing on early identification of academic struggles and tailored interventions. It is built on evidence-based principles from existing literature on academic failure and reflective practices. It intends to provide a structured approach to identify at-risk students and address academic failures, in its initial conceptual phase. This theoretical underpinning provides initial credibility until practical validation is feasible. Validation will follow pilot implementation to refine practicalities and identify unforeseen challenges. This limitation of our study offers food for thought for every institution to customize the framework to suit their local contexts while adhering to its core principles.

## Authors’ Contribution:

**RA, YR** and **AR:** Conceived the idea, designed the study, collected data, did thematic analysis, manuscript writing and final review.

All authors take full responsibility for the integrity of research therefore fulfilling all four ICMJE criteria for authorship.

## References

[1] Over 180,000 students take MDCAT exam (2023). Dawn [Internet].

[2] Bennion LD, Durning SJ, LaRochelle J, Yoon M, Schreiber-Gregory D, Reamy BV (2018). Untying the Gordian knot:Remediation problems in medical schools that need remediation. BMC Med Educ.

[3] Pinyopornpanish M, Sribanditmongkok P, Boonyanaruthee V, Chan-ob T, Maneetorn N, Uuphanthsath R (2004). Factors affecting low academic achievement of medical students in the faculty of medicine, Chiang Mai University. Changi Mai Med Bull.

[4] Kiran F, Javaid A (2020). Students'perceptions of factors for academic failure in pre-clinical years of a medical school. J Pak Med Assoc.

[5] Yates J (2011). Development of a 'toolkit'to identify medical students at risk of failure to thrive on the course:an exploratory retrospective case study. BMC Med Educ.

[6] Rashid A, Yasmeen R, Ahmed R, Jawed K (2022). Factors leading to the academic failure of undergraduate medical students - Predict early to prevent. Pak J Med Sci.

[7] Pangaro L, ten Cate O (2013). Frameworks for learner assessment in medicine:AMEE Guide No. 78. Med Teach.

[8] McMeekin N, Wu O, Germeni E, Briggs A (2020). How methodological frameworks are being developed:evidence from a scoping review. BMC Med Res Methodol.

[9] Hayat AA, Shateri K, Amini M, Shokrpour N (2020). Relationships between academic self-efficacy, learning-related emotions, and metacognitive learning strategies with academic performance in medical students:a structural equation model. BMC Med Educ.

[10] Morosanova VI, Bondarenko IN, Fomina TG (2022). Conscious Self-regulation, Motivational Factors, and Personality Traits as Predictors of Students'Academic Performance:A Linear Empirical Model. Psychol Russ.

